# The role of preoperative albumin and white blood cell count in surgical site infections following whipple surgery

**DOI:** 10.1038/s41598-022-21849-2

**Published:** 2022-11-10

**Authors:** Mohamad Ali Tfaily, Paola Ghanem, Sarah H. Farran, Fatema Dabdoub, Zeina A. Kanafani

**Affiliations:** 1 Department of Internal Medicine, Emory University, Atlanta, GA USA; 2 Department of Internal Medicine, American University of Beirut Medical Center, Faculty of Medicine, Beirut, Lebanon; 3 Department of Medical Oncology, Johns Hopkins University, Baltimore, MD USA; 4 Department of Pathology and Laboratory Medicine, American University of Beirut, Beirut, Lebanon

**Keywords:** Diseases, Infectious diseases, Microbiology, Bacteria

## Abstract

Whipple surgery is associated with a high risk of surgical sites infections (SSIs). Nutritional deficiency has been associated with an increased risk of wound infections. This study aims at exploring the role of preoperative albumin levels in predicting the risk of SSIs following Whipple surgery. A total of 23,808 individuals were identified from the ACS-NSQIP database from years 2011 to 2017. The primary exposure was pre-operative albumin while the secondary exposure was white blood cell (WBC) count. The primary outcome was divided into superficial and deep surgical site infections (S/D SSI) and organ-space SSI. All statistical analyses were conducted using IBM Statistical Package for Social Sciences version 26. Levels of pre-operative serum albumin less than 3.73 g/L, dirty and contaminated wounds and longer operative time were associated with increased odds for developing S/D SSIs (OR = 1.14, OR = 1.17, OR = 1.06, respectively, p-value < 0.05). Pre-operative WBC level (/L) was associated with a risk of developing an organ-space SSI but not S/D SSI (OR = 1.02, p-value  0.003). This study demonstrates the predictive role of pre-operative albumin in developing S/D SSIs and highlights the need to develop therapeutic strategies to optimize the pre-operative nutritional health status of patients undergoing Whipple surgery.

## Introduction

Pancreatic cancer is the sixth leading cause of cancer deaths worldwide, with up to 432,000 reported deaths in 2018, with more than 458,000 incident cases in 2018^1^. Owing to the advanced stage at the time of diagnosis, death rate due to pancreatic cancer is 12.6 per 100,000 in males and 9.6 per 100,000 in females^2^. Pancreatic cancer is predicted to become the second leading cause of cancer-related mortality by 2030 in the United States^3^. Whipple procedure, or pancreaticoduodenectomy, is used to resect these tumors, and allows the removal of the head of the pancreas and part of the duodenum, gallbladder, and biliary tree. Despite this intervention, the 5-year survival after pancreaticoduodenectomy in patients with node negative disease is only 30%, and only 10% in their node positive counterparts^4^. In addition, this procedure is associated with significant morbidity, with complication rates reaching 41%, prolonging hospital stay and increasing the rates of readmission^5^.

Surgical site infections (SSIs) are the most common complication following the surgical management of pancreatic cancer^6^. These can be superficial, only involving the skin, or deep, involving deeper structures like the fascia or muscles. They can also be organ-space based (Table 1). Depending on the cohort, SSIs have been reported to occur at variable rates ranging from 17 to 23.5% after Whipple surgery^6,7^. In addition, organ-space surgical site infections leading to intraabdominal abscess formation have been found to be among the leading causes of readmission in these patients, contributing up to 21.4% of all readmissions^7^. SSIs can generally be prevented by addressing the underlying risk factors. Identifying these risk factors for SSIs can provide optimal prophylactic management to improve post-operative outcomes and quality of life, decrease re-admission rate^8,9^ and minimize treatment cost by 23.8%^10^. Of the risk factors for surgical site infections (SSIs), hypoalbuminemia and elevated white blood cell counts (WBC) have been examined in populations and procedures as orthopedic surgery^11,12^. For Whipple procedure, however, these factors remain poorly studied with inconsistent evidence, likely due to small sample size of the available studies^13–21^. In this retrospective analysis of the American College of Surgeons-National Surgical Quality Improvement Program (ACS-NSQIP) database, we aim at exploring the role of preoperative albumin levels in predicting the risk of SSIs following Whipple surgery with partial or total resection.

**Table 1:** CDC definitions of different types of surgical site infections.

Superficial SSI	Infection involving the skin where the incision was made
Deep SSI	Infection beneath the incision area involving the soft tissue (muscle, connective tissue)
Organ-space SSI	Infection in any area or organ in the body involved in the surgery other than skin and soft tissue

*SSI* surgical site infection.

## Methods

### Data source

The ACS-NSQIP database from years 2011 to 2017 was used to test our research hypothesis. This externally validated database collects information from patients from more than 600 medical centers in the United States up to 30 days after undergoing surgical procedures^22^. The aim of the ACS-NSQIP database is to assess surgical outcomes to enhance surgical care^22^. This study uses deidentified patient data and is thus IRB-exempt. The authors agreed to and signed the data use agreement of the ACS NSQIP dataset in order to perform a retrospective analysis of deidentified patient data from the registry.

### Patient selection

Patients who underwent total or partial pancreaticoduodenectomy during the years 2011–2017 were identified using the current procedure terminology (CPT) codes: 48150, 48152, 48153 and 48154. All patients older than 18 years of age were included in this study. Patients without pre-operative albumin or without pre-operative WBC count were excluded from the study.

### Study covariates

Demographic characteristics (age, gender, race) and anthropomorphic data (height, weight, BMI) were obtained. Pre-existing comorbidities such as diabetes mellitus, heart failure, chronic kidney disease, presence of dyspnea, disseminated cancer, presence of ascites, and presence of sepsis prior to surgery were included as covariates. Surgical factors such as wound classification, total operation time, peri-operative bleeding, and whether the procedure was pylorus sparing were also recorded, as well as laboratory levels of pre-operative bilirubin, sodium, blood urea nitrogen, and hematocrit. Finally, other variables such as smoking status, steroid use, more than 10% body weight loss in the last 6 months, functional health status and the American Society of Anesthesiologists (ASA) physical status classification were studied.

Missing values for categorical variables (ASA status and transfusion status) were imputed based on the mode of each variable. For continuous variables, missing values for height were imputed based on the mean, conditioned by gender and age. Missing data for these covariates was rare and imputation did not exceed 5% of the values for each variable.

Our primary study variable was pre-operative albumin. It was statistically analyzed and inserted into the models as a binary variable, using the mean of 3.7. Effect modification was explored for several covariates that were suspected of having an interaction with albumin. We also explored the effect of pre-operative WBC count on the main outcome and recorded it as a secondary analysis.

### Study outcomes

The primary outcome was divided into superficial and deep surgical site infections (S/D SSI) on one hand and organ-space SSI on the other hand. Superficial and deep SSIs were recoded into one variable as they both share similar pathophysiology^23^. These study endpoints were defined as any SSI occurring within 30 days post-surgical procedure. Detailed description of each variable was extracted from the Participant Data Use file (2014).

### Statistical analyses

All statistical analyses were conducted using IBM Statistical Package for Social Sciences version 26 (IBM Corp, Armonk, NY). Descriptive analysis was performed and expressed as mean ± standard deviation for continuous variables and count (percent) for categorical variables. For multivariate analysis, variables were analyzed using forward logistic regression. Statistically or clinically significant variables from the bivariate analyses were entered in the multivariate analysis using a cut-off for two-tailed p-value of 0.2. Two final logistic regression models were computed: one with S/D SSIs as an outcome and another model with organ SSIs as an outcome with particular interest in pre-operative albumin and WBC count. All p-values obtained are from two-tailed tests.

### Ethics

This is an observational study using de-identified previously collected patient data and no ethical approval is required.

### Accordance statement

All methods were performed in accordance with the relevant guidelines and regulations. This study was exempt from the American University of Beirut Institutional Review Board as the ACS-NSQIP does not provide protected health information from individuals.

## Results

### Study population and baseline characteristics

The total sample size consisted of 23,808 patients who met the eligibility criteria from the NSQIP database (2011–2017). Table 2 shows the baseline characteristics of the sample including demographics, comorbidities, and perioperative and surgical factors. The mean age in the patient population was 65 $\pm$ 12 years, with 53.7% of patients being male, and a mean BMI of 27.2 ± 5.8 kg/m^2^. Smoking within a year of surgery was recorded in 19.7% of the cohort. The vast majority of patients (98.9%) were functionally independent prior to surgery. A total of 26% of the patients in the sample were diabetic on therapy with either an oral agent or insulin therapy (12.8% and 13.2%, respectively), while 54.3% had hypertension requiring pharmacologic treatment. A non-pylorus sparing procedure was performed in 61.7% of patients. 45.1% of the patients had an albumin level below 3.73 g/L. 10.8% developed S/D SSIs, while 14.2% developed organ SSIs.

**Table 2:** Demographic characteristics of the cohort.

Variable	Mean (SD)	n (%)
Age^a^ (years)	65 (12.0)	
BMI (kg/m^2^)	27.2 (5.8)	
Male gender		12,787 (53.7)
Current smoker within one year		4692 (19.7)
**Functional health status prior to surgery**		
Independent		23,550 (98.9)
Partially dependent		233 (1.0)
Totally dependent		25 (0.1)
**DM with oral agents or insulin**		
Insulin		3140 (13.2)
Non-insulin		3055 (12.8)
CHF 30 days before surgery		91 (0.4)
Hypertension requiring medication		12,922 (54.3)
Dyspnea		1,399 (5.9)
History of severe COPD		1,058 (4.4)
Ascites		92 (0.4)
Acute renal failure (post-operatively)		24 (0.1)
Currently on dialysis (pre-operatively)		76 (0.3)
Disseminated cancer		1,104 (4.6)
Open wound infection		152 (0.6)
Steroid use for chronic condition		640 (2.7)
> 10% weight loss in last 6 months		3,940 (16.5)
Bleeding disorders		651 (2.7)
Transfusion ≥ 1 unit PRBCs within 72 h before surgery		305 (1.3)
Systemic sepsis		386 (1.6)
Pre-operative serum sodium (mmol/L)	138.8 (3.3)	
Pre-operative BUN (mg/dL)	15.1 (7.3)	
Pre-operative serum creatinine (mg/dL)	0.9 (0.4)	
Pre-operative serum albumin (g/dL)	3.7 (0.6)	
Pre-operative total bilirubin (mg/dL)	1.79 (2.7)	
Pre-operative AST (U/L)	55.6 (72.5)	
Pre-operative alkaline phosphatase (U/L)	190.9 (172.4)	
Pre-operative WBC (1/m^3^)	7.4 (2.8)	
Pre-operative hematocrit (%)	37.4 (5.1)	
Pre-operative platelet count × 10^3^/mL	261.1 (94.3)	
Pre-operative PTT	21.2 (13.7)	
Pre-operative INR	1.0 (0.2)	
**Wound classification**		
Clean-contaminated		20,102 (84.4)
Contaminated		2996 (12.6)
Dirty infected		710 (3.0)
**ASA classification**		
Class 1		107 (0.4)
Class 2		5273 (22.1)
Class 3		16,828 (70.7)
Class 4		1595 (6.7)
Class 5		5 (0.0)
Pylorus sparing		9129 (38.3)
Total number of patients (N)		23,808

*SD* standard deviation, *BMI* body mass index, *DM* diabetes mellitus, *CHF* congestive heart failure, *COPD* chronic obstructive pulmonary disease, *pRBC* packed red blood cells, *BUN* blood urea nitrogen, *HCT* hematocrit, *WBC* white blood cells, *AST* aspartate transaminase, *PTT* partial thromboplastin time, *INR* international normalized ratio, *ASA* American Society of Anesthesiologists.

^a^With patients over 89 coded as 90+.

The bivariate and multivariate associations between S/D SSIs and variables found significant in the regression analysis are displayed in Table 3. After adjusting for other variables, pre-operative serum albumin lower than 3.73 g/L was significantly associated with an increased risk of developing S/D SSIs (OR = 1.14, 95% CI 1.05–1.24; p-value = 0.002). Other risk factors independently associated with S/D SSIs included smoking within a year of surgery (OR = 1.20, p-value =  < 0.001), having dyspnea (OR = 1.30, p-value < 0.001), having a bleeding disorder (OR = 1.35, p-value = 0.009), a dirtier wound classification compared to a clean-contaminated wound (OR = 1.17, p-value < 0.001) and a longer operative time (OR = 1.06, p-value < 0.001). An increased pre-operative WBC level was not statistically associated with developing a S/D SSI.

**Table 3:** Bivariate and multivariate regression analysis results for the for the risk factors of superficial and deep SSIs.

Variables	uOR	95% CI for uOR	aOR	95% CI for aOR	p-value for aOR
Age^a^ (years)	1.00	0.99–1.00	−	−	−
BMI (kg/m^2^)	1.02	1.01–1.03	1.02	1.01–1.03	< 0.001
Smoking within a year of surgery	1.18	1.07–1.30	1.20	1.08–1.32	< 0.001
Hypertension	1.05	0.97–1.14	−	−	−
DM	0.96	0.87–1.05	0.89	0.81–0.98	0.013
Steroid use for chronic condition	1.2	0.97–1.48	−	−	−
Dyspnea	1.41	1.21–1.65	1.30	1.11–1.53	< 0.001
Bleeding disorder	1.38	1.10–1.72	1.35	1.08–1.94	0.009
Serum albumin < 3.73 (g/dL)	1.17	1.08–1.27	1.14	1.05–1.24	0.002
WBC count (1/m^3^)	1.01	1.00–1.03	−	−	−
Wound classification (clean-contaminated)	1.21	1.11–1.31	1.17	1.08–1.27	< 0.001
Operative time (hours)	1.07	1.05–1.09	1.06	1.04–1.08	< 0.001

Variables dropped from the model due to insignificance at the multivariate level: steroid use for chronic condition, hypertension and age.

*SSI* surgical site infection, *uOR* unadjusted odds ratio, *aOR* adjusted odds ratio, *CI* confidence interval, *BMI* body mass index, *DM* diabetes mellitus, *WBC* white blood cells.

^a^With patients over 89 coded as 90+.

Table 4 shows the bivariate and multivariate associations between organ-space SSIs and variables found significant in the regression analysis. After adjusting for other variables, WBC count (OR = 1.02, 95% CI 1.01–1.03; p-value = 0.012) was significantly associated with developing an organ-space SSI, while pre-operative albumin levels did not significantly affect the odds of this outcome. Smoking (OR = 0.86, p-value = 0.002), weight loss of > 10% in the last 6 months (OR = 0.85, p-value = 0.004), and undergoing a pylorus-sparing procedure (OR = 0.89, p-value = 0.002) significantly reduced the odds of developing an organ-space SSI. On the other hand, belonging to a racial minority (OR = 1.30, p-value < 0.001), having disseminated cancer (OR = 1.29, p-value = 0.002), a bleeding disorder (OR = 1.40, p-value = 0.001), systemic sepsis (OR = 1.36, p-value = 0.027), and a longer operative time (OR = 1.04, p-value < 0.001) were all associated with increased odds for developing an organ-space SSI after adjusting for other variables.

**Table 4:** Bivariate regression and multivariate regression analysis results for the risk factors of organ-space SSIs.

Variables	uOR	95% CI for uOR	aOR	95% CI for aOR	p-value for aOR
Age^a^ (years)	1.00	0.99–1.00	−	−	−
Female	0.74	0.69–0.80	0.79	0.73–0.85	< 0.001
BMI (kg/m^2^)	1.03	1.03–1.04	1.04	1.03–1.04	< 0.001
Racial minority	0.85	0.78–0.92	1.30	1.19–1.42	< 0.001
DM	0.86	0.79–0.93	0.79	0.73–0.87	< 0.001
Steroid use for chronic disease	1.2	0.97–1.48	−	−	−
Hypertension	1.10	1.02–1.18	−	−	−
Smoking	0.83	0.75–0.91	0.86	0.77–0.94	0.002
Disseminated cancer	1.30	1.10–1.52	1.29	1.10–1.52	0.002
> 10% weight loss in last 6 months	0.76	0.68–0.84	0.85	0.77–0.95	0.004
Bleeding disorders	1.40	1.14–1.71	1.40	1.14–1.72	0.001
Systemic sepsis	1.40	1.08–1.81	1.36	1.04–1.78	0.027
Serum albumin < 3.73 (g/dL)	1.10	1.02–1.19	−	−	−
Serum sodium (mmol/L)	1.03	1.02–1.04	1.02	1.01–1.03	0.002
Serum BUN (mg/dL)	1.01	1.00–1.01	1.01	1.00–1.01	0.02
WBC count (/m^3^)	1.02	1.01–1.03	1.02	1.01–1.03	0.012
HCT (%)	1.03	1.02–1.04	1.03	1.02–1.03	< 0.001
Wound classification (clean-contaminated)	1.20	1.11–1.29	1.20	1.11–1.29	< 0.001
Operative time (hours)	1.00	1.00–1.00	1.04	1.02–1.06	< 0.001
Pylorus sparing procedure	0.85	0.79–0.91	0.91	0.85–0.98	0.011

Variables dropped from the model due to insignificance at the multivariate level: preoperative serum albumin, steroid use for chronic condition, age and hypertension.

*SSI* surgical site infection, *uOR* unadjusted odds ratio, *aOR* adjusted odds ratio, *CI* confidence interval, *BMI* body mass index, *DM* diabetes mellitus, *BUN* blood urea nitrogen, *HCT* hematocrit, *WBC* white blood cells.

^a^With patients over 89 coded as 90+.

## Discussion

This study analyzed the role of pre-operative albumin and white blood cell count, among other covariates, in predicting the occurrence of surgical site infection after Whipple surgery using the General Surgery NSQIP dataset from 2011 through 2017. For S/D SSI, having a serum albumin < 3.73 g/L was found to significantly increase the odds of developing the SSI by 14%. For organ-space SSI, pre-operative serum albumin was not significantly associated with the outcome.

Serum albumin is an indicator of a patient’s nutritional and inflammatory status^24^. In in vivo models, hypoalbuminemia was associated with poor tissue healing, decreased collagen synthesis and granuloma formation in surgical wounds^25–27^. The suggested mechanism through which albumin affects wound healing is by upregulating the transcription of tissue-forming proteins that activate the NFKB pathway, thus enhancing the healing process^28^. Hypoalbuminemia, on the other hand, can increase the expression of TNF-ɑ , C-Reactive Protein (CRP) and several interleukins, all known to be acute-phase proteins that promote tissue damage^29,30^.

There is evidence form the literature that patients with hypoalbuminemia have 2.9 times the odds of developing a major wound complication after vulvectomy^31^, and twice the risk after total joint arthroplasty^32^. It has also been associated with delay in wound healing following abdominoperineal resection^33^. A Cochrane review of 13 randomized-controlled trials found that pre-operative nutritional optimization through parenteral nutrition decreased overall complications rates following gastrointestinal surgeries, and “immune enhancing” drinks decreased infectious complications including wound infections and abdominal abscesses from 27 to 14%^34^ The role of albumin and other indicators of nutritional status in the prevention of post-surgical infectious complications is increasingly being studied^35^. SSIs following several types of surgeries^32,36–41^ have been reported in patients with pre-operative hypoalbuminemia. In patients undergoing surgeries for pancreatic cancer in specific, evidence points out to the increased morbidity from all causes due to overall malnutrition and hypoalbuminemia^42,43^. A meta-analysis examining the risk factors for SSIs after pancreatic surgery did not show a significant role of albumin in SSI^44^. The findings in this study are limited by the fact that S/D and organ-space SSIs were combined into one outcome of interest. However, the different pathophysiology of S/D and organ-space SSIs, warrants the differential investigation of clinical risk factors of the separate types of SSIs.

While the results of our study regarding albumin are in harmony with the findings above when it comes to S/D SSI, we were not able to reproduce the same results with organ-space SSI. The literature remains inconsistent regarding the role of pre-operative albumin in predicting organ-space SSI. A recent retrospective study performed on patients undergoing pancreatic surgery showed no significant association between preoperative albumin levels and the development of organ-space SSI^45^ while in another study, hypoalbuminemia was predictive of an increased risk of organ-space SSI^46^.

Evidence on the role of pre-operative WBC count in the development of SSI has also been debated. In a study on patients undergoing cardiac surgery, pre-operative leukocytosis was not associated with superficial or organ-space SSI^47^, while other studies have found a significant correlation between pre-operative leukocytosis and postoperative infectious complications^48^. This leukocytosis may be caused by a pre-existing infection, posing a higher risk of SSI after surgery. It could also be associated with malnutrition or other risk factors that render the individual at higher risk of SSI^48^. However, there are limited data on the correlation between asymptomatic leukocytosis and SSI in patients undergoing Whipple surgery. Our results indicate a positive association between increased WBC count and organ-space SSI but not S/D SSI.

A > 10% loss in body weight in the 6 months preceding surgery was associated in this study with decreased odds of developing organ-space SSIs. One survival analysis conducted on a smaller cohort of pancreatic cancer patients linked > 10% weight loss to worse disease outcomes, while another established that weight loss alone was not a prognostic factor^49^. A possible explanation of this finding could be the absence of staging variables in NSQIP that would adjust for cancer-related weight loss versus healthy-intended weight loss, especially that obesity is an established risk factor for several types of malignancies, including pancreatic carcinomas^50–52^. In addition, higher BMI was associated with higher risk of SSIs in our study, as well as several other studies in the literature^53^.

An unexpected finding in our study is the protective effect of smoking for organ/space SSIs. Smoking is a well-established risk factor for SSIs^54–56^. The seemingly protective effect could possibly be explained by a survival bias (collider bias), whereby healthy, younger patients would continue smoking while those with more comorbidities would discontinue smoking. This is particularly plausible since NSQIP assesses smoking within a year of surgery rather than lifetime smoking. Similarly, DM was found to be associated with a decreased risk of organ SSI, despite the well-known risk DM poses on infection^57^. We believe that several patients may have had undiagnosed DM, given the strong association between advanced pancreatic carcinoma and DM development^58–60^. In our cohort, nondiabetics were more likely to have disseminated cancer, further justifying this finding.

This study has several strengths and limitations. The ACS NSQIP used in the study provided a large sample size and high statistical power. Unfortunately, the dataset lacks data relevant to pancreatic surgery including chemotherapy/radiotherapy administration, pre-operative cholangitis and preoperative biliary stenting. In addition, the appropriateness of preoperative prophylactic antibiotics is a crucial confounder that is not provided by NSQIP, and this could represent a limitation^61^. To overcome this limitation, we assumed all patients received the standard antibiotic prophylaxis according to the available guidelines. Additional challenges are inherent to the database in that despite being large, it depends on the nature and comprehensiveness of data provision by each institution^45^. To circumvent these challenges, we assumed that the values missing in this dataset were missing at random. We excluded patients who had missing values for albumin and WBC count, the primary and secondary exposures respectively, and we imputed the remaining missing values using the median and mode as previously described.

## Conclusion

Despite available evidence, strategies to optimize pre-operative nutrition remain inadequately developed. Moreover, available guidelines for the prevention of surgical site infections do not, thus far, include clear recommendations regarding the role of controlling inflammatory and nutritional markers to prevent post-operative infections, especially in this specific patient population^62^. This study essentially provides further evidence in favor of pre-operative health management and nutritional optimization in patients undergoing Whipple surgery, a population at high risk of morbidity and mortality, with special attention to pre-operative albumin and WBC count.

Novel studies are needed to develop treatment algorithms to optimize the pre-operative health status of patients undergoing Whipple surgery. This study reaffirms the role of albumin in wound healing and adds to the building evidence on the role of preoperative albumin and WBC count in surgical site infections.

## Data Availability

The dataset analyzed during the current study is available by request on the website of the American College of Surgeons (https://www.facs.org/quality-programs/acs-nsqip). However, the used datasets are available from the corresponding author on reasonable request.
